# P-1747. Optimizing Candidaemia Management: Antifungal Stewardship and Diagnostic Practices in a UK Tertiary Centre

**DOI:** 10.1093/ofid/ofaf695.1918

**Published:** 2026-01-11

**Authors:** Sajeevan Rasanantham, Heather Dolby

**Affiliations:** South Tees NHS Foundation trust, Middlesbrough, England, United Kingdom; James Cook University Hospital, Middlesbrough, England, United Kingdom

## Abstract

**Background:**

Candidaemia is associated with high mortality and increasing incidence, particularly in critical care and post-surgical settings. Guideline-concordent management, including prompt antifungal therapy, repeat cultures, source control, and investigation for metastatic complications, is essential for improving outcomes. This study evaluated real-world practice patterns in antifungal use, microbiological monitoring in a UK teaching hospital.

**Figure:**
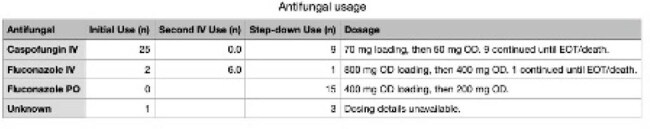
Summary of antifungal usage Summary of antifungal usage and dosage

**Figure:**
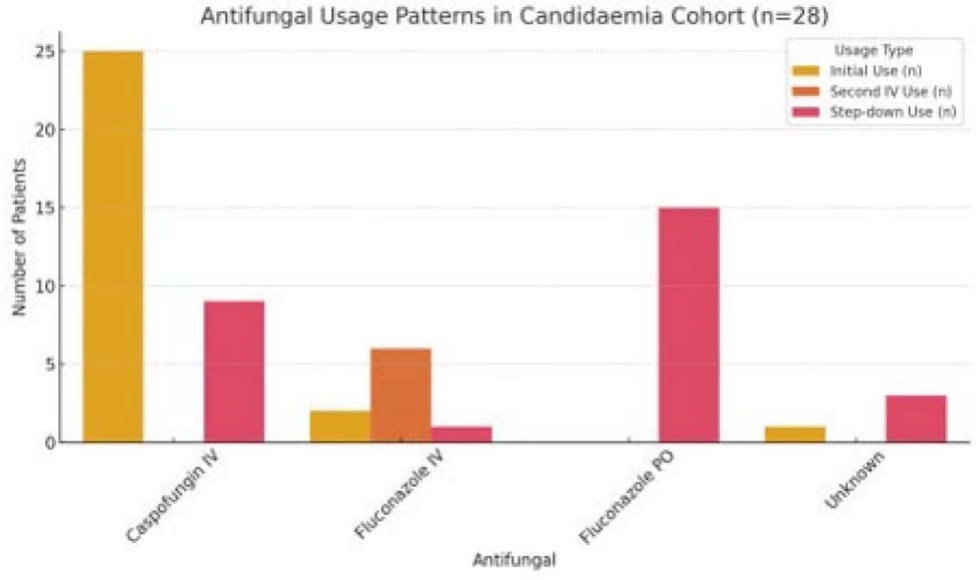
Antifungal usage Patterns in Candidaemia cohort Antifungal usage Patterns in Candidaemia cohort

**Methods:**

A retrospective review was conducted of all inpatients with candida species positive blood cultures between June 1, 2022 and June 1, 2023. Data from the laboratory information Management system and case notes were analysed for organism identification with susceptibility, treatment regimens and microbiological follow-up.

**Figure:**
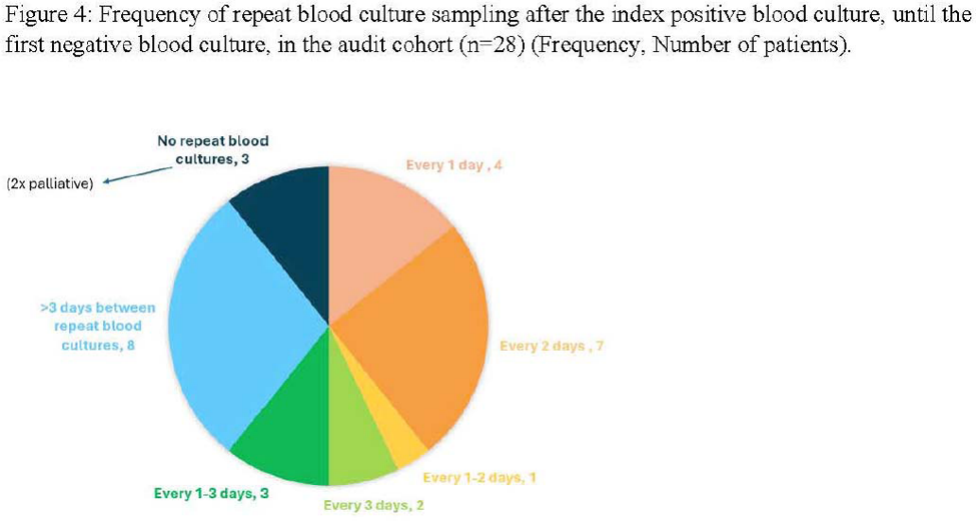
Frequency of repeat blood cultures Frequency of repeat blood cultures: Number of days

**Results:**

Twenty-eight patients met inclusion criteria. Species identification was successful in all cases; susceptibility testing was performed in 96.4%. The most common species were *C.albicans(50%), C.glabrata( 28.6%), and C.parapsilosis*(21.4%). Repeat blood cultures were taken in 46.2% of non-palliative patients(see figure 1), with a median clearance time of 3 days. Antifungal therapy was appropriately initiated in 96.4% of patients, with 89.5% receiving treatment for a guideline-recommended duration of (median 19 days). Caspofungin was the initial agent in 89.3% of cases; Fluconazole step-down therapy( IV to Oral) was used in 60.7% of patients(see table 1).

**Conclusion:**

This single-centre study demonstrates strong adherence to antifungal prescribing guidlines and effective diagnostic stewardship. Nonetheless, improvements are needed in the consistency of repeat blood cultures. Optimising antifungal and diagnostic stewardship areas may further reduce morbidity and mortality in patients with candidaemia.

**Disclosures:**

All Authors: No reported disclosures

